# Mixotrophy in Chlorophytes and Haptophytes—Effect of Irradiance, Macronutrient, Micronutrient and Vitamin Limitation

**DOI:** 10.3389/fmicb.2018.01704

**Published:** 2018-07-31

**Authors:** Ruth Anderson, Sophie Charvet, Per J. Hansen

**Affiliations:** ^1^Marine Biology Section, Department of Biology, University of Copenhagen, Helsingør, Denmark; ^2^Leibniz Institute for Baltic Sea Research Warnemuende, Rostock, Germany; ^3^Biology and Paleo Environment, Lamont-Doherty Earth Observatory, Columbia University, New York, NY, United States

**Keywords:** mixotrophy, phytoflagellate, haptophyte, chlorophyte, bacterivory, mixotrophic growth, green algae

## Abstract

Chlorophytes and haptophytes are key contributors to global phytoplankton biomass and productivity. Mixotrophic bacterivory has been detected for both groups, but a shortage of studies with cultured representatives hinders a consistent picture of the ecological relevance and regulation of this trophic strategy. Here, the growth, primary production, fraction of feeding cells (acidotropic probes) and bacterivory rates (surrogate prey) are tested for two species of the chlorophyte genus *Nephroselmis* and the haptophyte *Isochrysis galbana* under contrasting regimes of light (high vs. low) and nutrients (non-limited and macronutrient-, micronutrient- and vitamin-limited), at low bacterial concentrations (<10^7^ bacteria mL^−1^). All three species were obligate phototrophs, unable to compensate for low light conditions through feeding. Under nutrient limitation, *N. rotunda* and *I. galbana* fed, but growth ceased or was significantly lower than in the control. Thus, mixotrophic bacterivory could be a survival rather than a growth strategy for certain species. In contrast, nutrient-limited *N. pyriformis* achieved growth rates equivalent to the control through feeding. This strikingly differs with the classical view of chlorophytes as primarily non-feeders and indicates mixotrophic bacterivory can be a significant trophic strategy for green algae, even at the low bacterial concentrations found in oligotrophic open oceans.

## Introduction

Mixotrophy, considered here as the combined use of photosynthesis and prey phagocytosis to meet nutritional and energetic requirements, is now recognized as a global, environmentally relevant trophic strategy for small (<20 μm) phytoflagellates (Hartmann et al., 2012; Mitra et al., 2013). Mixotrophic bacterivory can account for 50–95% of bacterial losses due to predation in marine systems (Unrein et al., 2007; Zubkov, and Tarran, 2008; Anderson et al., 2017), and could impact trophic transfer efficiency to higher levels in the food chain, leading to larger mean organism sizes (Ward and Follows, 2016). Bacterial phagocytosis has been found for most major phytoflagellate phylogenetic groups and across a wide range of aquatic habitats (Schmidtke et al., 2006; Unrein et al., 2013; McKie-Krisberg et al., 2015; Yoo et al., 2017). This genetic and ecological heterogeneity additionally translates into a large functional and trophic diversity, with tested species varying in how feeding is regulated, mixotrophic growth efficiency and whether prey is utilized to obtain both nutrients and biomass for growth (Terrado et al., 2017) or primarily nutrients (Carvalho and Granéli, 2010). Despite this, studies assessing feeding and mixotrophic growth in cultured small phytoflagellate strains are scarce and unevenly distributed across phylogenetic groups. As an example, more studies exist on the chrysophyte genus *Ochromonas* than on the entire division Chlorophyta. This imbalance in studied species is not always based on marine environmental relevance (e.g., *Ochromonas* spp. generally does not play a major role in marine ecosystems, but it was one of the first genera where mixotrophy was described and the focus of much of the early work on the topic). Thus, filling this knowledge-gap is critical to understanding the regulation and magnitude of mixotrophy in marine systems, and thereby its impact on ecosystem productivity (Mitra et al., 2014).

The present study is focused on chlorophytes and haptophytes. Both are globally distributed and extremely diverse phylogenetic groups, which significantly contribute to marine phytoplankton biomass and primary production (Worden et al., 2004; Liu et al., 2009; Jardillier et al., 2010). Haptophytes have been shown to be major bacterivores in marine systems (Frias-Lopez et al., 2009; Unrein et al., 2013) and bacterial ingestion has been detected for a number of genera (Table 1). However, the majority of culture studies on mixotrophy for this group have focused on the relatively large (normally >5 μm in diameter) *Prymnesium parvum* and *Chrysochromulina* spp., which tend to have a broad prey spectra, consuming both bacteria and other protists (Hansen and Hjorth, 2002; Tillmann, 2003). So far, for haptophytes within the size range determined to be important bacterivores in the field (3–5 μm; Unrein et al., 2013), there are very few studies assessing bacterial ingestion and no studies on mixotrophic growth (Table 1). For chlorophytes, the first instance of bacterivory was not recorded until the 1990s, when digestive activity was detected for *Micromonas pusilla* (Gonzalez et al., 1993). Since then, phagotrophy has only been confirmed for 5 additional species (Table 1). Clear indications exist for an important role of bacterivory for chlorophytes in polar regions (McKie-Krisberg and Sanders, 2014; McKie-Krisberg et al., 2015), but whether this is also the case for temperate strains and environments remains to be elucidated.

**Table 1:** Chlorophyte and haptophyte genera for which bacterial ingestion (B) and/or mixotrophic growth (G) has been tested under different regimes of light (dark or low light vs. high light) and nutrients [low nutrient concentrations (LN), high nutrient concentrations (HN) or field samples with unknown nutrient concentrations (F)].

	**Species**	**Habitat**	**Dark/Low light**			**High light**			**Primary carbon source for growth**	**References**
			**LN**	**HN**	**F**	**LN**	**HN**	**F**		
**CHLOROPHYTA**										
*Mantoniella*	sp.	MT					-B			Anderson et al., 2017
	*antarctica*	MP	B^G^	B		B^G^	B			Gast et al., 2014; McKie-Krisberg and Sanders, 2014
*Pyramimonas*	*mitra*	MT					-B			Anderson et al., 2017
	*disomata*	MT					-B			Anderson et al., 2017
	*melkonianii*	MT					-B			Anderson et al., 2017
	*tychotreta*	MP	B^G^	-B		B^G^	-B			Gast et al., 2014; McKie-Krisberg and Sanders, 2014
	*gelidicola*	FP			B			B		Bell and Laybourn-Parry, 2003
*Nephroselmis*	*rotunda*	MT		-B/-G		B^Mi^/G^Mi^	B		PP	Present study; Anderson et al., 2017
	*pyriformis*	MT		-B/-G		B/G^Ma^^Mi^^V^	B		PP	Present study; Anderson et al., 2017
*Cymbomonas*	*tetramitiformis*	MT		B/-G			-B			Maruyama and Kim, 2013; Burns et al., 2015
*Micromonas*	*pusilla*	MT					B			Gonzalez et al., 1993
	spp.	MT						-B		Unrein et al., 2013
	sp.	MP	B^a^	B	B	B^G^	B			McKie-Krisberg and Sanders, 2014
*Pycnococcus*	*provasolli*	MT					-B			Gonzalez et al., 1993
*Brachiomonas*	*submarina*	MP		-B						Jochem, 1999
**HAPTOPHYTA**										
*Prymnesium*	*parvum*	MT		B/-G		B/G^GP^	B/G		PP	8 - Legrand et al., 2001; Liu et al., 2016
*Chrysochromulina*	*ercinia*	MT		B		B/G^P^	B/G	B	PP	Havskum and Riemann, 1996; Safi and Hall, 1999; Hansen and Hjorth, 2002
	*simplex*	MT					-B			Anderson et al., 2017
	*brevifilium*	MT					-B			Anderson et al., 2017
	*hirta*	MT		B			B			Kawachi, 1991; Jochem, 1999
	*minor*	MT						B		Havskum and Riemann, 1996
	*brachycylindricum*	MT						B		Havskum and Riemann, 1996
	spp.	MT						B		Epstein and Shiaris, 1992; Safi and Hall, 1999; Frias-Lopez et al., 2009
	*polylepsis*	FT				B^e^	B			Nygaard and Tobiesen, 1993
*Isochrysis*	*galbana*	MT		-B/G		B/G^Ma^^V^	B		PP	Present study
*Coccolithus*	*baraudi*	MT				B^G^				Houdan et al., 2006
*Imantonia*	spp.	MT					B/-B	B		Safi and Hall, 1999; Anderson et al., 2017; Anderson, unpublished data
*Pavlova*	*lutheri*	MT		-B						Jochem, 1999
	sp.	FT				B^P^				Nygaard and Tobiesen, 1993
*Apedinella*	*radians*	MT					B			Anderson, unpublished data
*Phaeocystis*	*antarctica*	MP			-B			-B		Moorthi et al., 2009

A (-) symbol indicates no bacterivory (-B) or no mixotrophic growth (-G) were detected. For many studies, only bacterial ingestion was measured but not mixotrophic growth (no G shown). For those studies where it was assessed, the postulated primary source of carbon for growth for the phytoflagellate species is given (primary production (PP) vs. uptake from prey). Habitats – MT, marine temperate; MP, marine polar; FT, freshwater temperate; FP, freshwater polar. For species where a large number of studies have been conducted the number of studies is given and 2 references are listed as examples.

G general, undefined, nutrient limitation;

Ma macronutrient limitation;

Mi micronutrient limitation;

V vitamin limitation;

P *P limitation*.

Another important understudied aspect is the influence of different regulating factors on mixotrophic feeding and growth. Irradiance levels and the availability of nutrients, complex macromolecules and prey have all been shown to be triggers for feeding among phytoflagellates (Kimura and Ishida, 1985; Skovgaard et al., 2003; McKie-Krisberg et al., 2015). However, the regulatory effects and interplay between these factors are complex and differ between tested species. For example, under light-limiting conditions, different phytoflagellate species have been observed to (i) feed and grow heterotrophically, such as certain species of the chrysophyte genus *Ochromonas* (Liu et al., 2016); (ii) show short-term feeding allowing for survival but not mixotrophic growth, such as strains of the haptophyte *Prymnesium parvum* (Liu et al., 2016); or (iii) cease to feed, such as the haptophyte *Pavlova* sp. (Jochem, 1999). To understand this complexity, a comprehensive view is needed on the influence of different regulating parameters on mixotrophic growth, but this is available for very few species. In addition, the regulatory effect of growth factors such as micronutrients and complex macromolecules remains almost untested (Table 1).

In the present study we compared, for the first time, the growth, primary production and feeding of two temperate species of the chlorophyte genus *Nephroselmis* and the small (4–5 μm in diameter) haptophyte *Isochrysis galbana* under contrasting light (high vs. low) and nutrient regimes (macronutrient, micronutrient and vitamin limitation). All three species were isolated from marine environments, and members of both genera have been repeatedly isolated or detected in geographically disperse marine environments pointing to a global distribution (e.g., Massana et al., 2004; Hu et al., 2016; Tragin et al., 2016). To our knowledge this constitutes the first study assessing the mixotrophic growth of chlorophytes under nutrient limiting conditions (Table 1); and one of the few studies assessing the effect of specific nutrient limitation on bacterial phagocytosis and mixotrophic growth of cultured algal strains in general.

## Materials and methods

### Algal strains and experimental conditions

Experiments were carried out with bacterized cultures of *Nephroselmis pyriformis* K0557, *Nephroselmis rotunda* K0556 and *Isochrysis galbana* K1355 (Norwegian Culture Collection for Algae). The strains are routinely maintained in f/2 media (Guillard and Ryther, 1962). Briefly, this media consists of natural sea water (here Øresund nutrient-limited deep-water) heated to 105°C for 20 min, to which 3 solutions are added: macronutrients (nitrate, phosphate and silicate), micronutrients (iron, copper, molybdenum, zinc, cobalt and manganese) and vitamins (B_1_, B_12_, and H) (see reference for exact composition). Tests were conducted prior to the experiments for each algal species to determine an optimal light intensity for growth (data not shown) and the pH range tolerated (Supplementary Figure 1). To deplete background nutrient concentrations in the cultures, the algal strains were repeatedly grown in f/20 media (10 x dilution of f/2 media) until dense at the selected experimental light intensity (250 μmol photons m^−2^ s^−1^), and transferred to fresh f/20 media. This step was repeated at least 3 times for each strain.

Experiments were carried out for each algal strain in a climate chamber at 15°C with a light: dark period of 14:10. A control (Ctl: full growth media [f/4 (2 × dilution of f/2 media) and high light (250 μmol photons m^−2^ s^−1^)] was compared to 4 treatments: (1) NMa: media without the addition of macronutrients and high light; (2) NMi: media without the addition of micronutrients and high light; (3) NV: media without the addition of vitamins and high light; and (4) LL: low light (5 μmol photons m^−2^ s^−1^) and full media (Supplementary Figure 2). For each treatment, 600 mL incubations were carried out in triplicate in tissue culture flasks for 7–8 days, with a starting algal concentration of ~ 3 × 10^3^ cell mL^−1^. Care was taken that at least 200 mL of culture volume remained at the end of the incubation period. For *I. galbana*, 20 mL subsamples of each treatment were further maintained under the respective experimental conditions until day 10. Samples for diverse parameters were taken at regular intervals as described below (Table 2). The pH in each experimental flask was monitored using a pH-meter (pH3210, WTW GmbH Germany). For media N and P concentrations, 20 mL subsamples were filtered through 0.2 μm syringe filters (VWR International, Denmark), and stored at −20°C until subsequent analysis on a Seal Analytical® Autoanalyzer, model AA3HR according to Koroleff (1970) and Solorzano and Sharp (1980). For *N. rotunda* three experiments were carried out, the first (Exp. 1) only tracked changes in some parameters and did not include LL; the second (Exp. 2) tracked all parameters for all treatments, but problems were encountered while measuring the bacterivory rates; and, therefore, a third reduced experiment (Exp. 3) was carried out with Ctl, NMa and NMi (Table 2).

**Table 2:** Summary of the measurements carried out in the different experiments.

	**N. rotunda Exp. 1**	**N. rotunda Exp. 2**	**N. rotunda Exp. 3**	***N. pyriformis***	***I. galbana***
Protist abundance	Daily	Daily	Day 0 and 6	Daily	Daily
Bacterial abundance		Daily	Day 0 and 6	Daily	Daily
pH	Daily	Daily	Day 0 and 6	Daily	Daily
Nutrients		Day 0 (sea-water), 3 (NMa) and 7 (all treatments)		Day 0 (sea-water), 3 (NMa) and 7 (all treatments)	Day 0 (sea-water), 3 (NMa) and 7 (all treatments)
Percentage of feeding cells	Daily	Daily	Day 0 and 6	Daily	Daily
Bacterivory rates			Day 6	Day 6	Day 6
Chl a concentration		Day 6		Day 6	Day 6
Primary production		Day 6		Day 6	Day 6
Cellular carbon content		Day 7		Day 7	Day 7

### Follow up experiments

The effect on feeding and growth of adding the respective “limiting” substrate to each treatment was assessed on day 7 for *N. pyriformis* NMa, NMi and NV; and *I. galbana* NMa and NV. Duplicate 20 mL flasks were prepared from each replicate. The “limiting” substrate solution was added to one flask (Addition 1; e.g., the vitamin solution to NV) while the other was left unamended. All flasks were incubated for 3 days under the experimental conditions described above, after which samples were taken for protist abundance, percentages of feeding cells and pH. For *N. pyriformis*, a third flask was prepared for each replicate, to which both “non-limiting” solutions were added (Addition 2; e.g., the macro- and micronutrient solutions to NV). Finally, the persistence of *N. pyriform*is feeding cells after limitation had ceased was assessed over 10 days after transferring a starved culture (grown until very dense in f/20 media) to fresh f/2 media.

### Flow cytometric determination of bacterial and phytoflagellate abundance and percentages of feeding cells

Phytoflagellates and bacteria were quantified on a FACS Canto II flow cytometer using a low and medium flow rate respectively, calibrated with TrueCount beads (all BD Biosciences). Phytoflagellates were quantified live and distinguished from background noise through differences in pigment fluorescence, side scatter (SSC) and forward scatter (FSC) (Supplementary Figure 3). The accuracy of the flow cytometer was tested for each algal strain by cross-comparison to Lugols' iodine solution-fixed samples (f.c. 2%) enumerated via light microscopy. Bacteria were quantified according to Gasol and del Giorgio (2000). The percentage of phytoflagellate cells containing food vacuoles, and therefore assumed to be feeding, was determined using the acidotropic probe LysoTracker Green DND-26 (LyT G) (ThermoFisher Scientific) as described in Anderson et al. (2017). No effect on measurements of non-specific binding of the probe has been detected following this protocol for the strains employed here and a for a wide range of phylogenetically diverse phytoflagellates in the size range of the species tested in the current study (Supplementary Figure 4; Anderson et al., 2017).

### Determination of bacterivory rates

A slightly modified version of the protocol from Sherr et al. (1987) was employed. Briefly, fluorescently labeled bacteria (FLB) were prepared from carbon-starved *Photobacterium angustum* (Anderson et al., 2017). 50 mL vials were prepared from each experimental flask and FLB were inoculated to 10–15% of the naturally co-occurring bacterial abundance. Samples were taken immediately upon FLB addition and after 10, 20, and 30 min incubation under the respective experimental conditions. Samples were fixed v/v with 4% very cold glutaraldehyde (f.c. 2%) (Sanders et al., 1989) and stored cool and in the dark between 2 and 24 h. Subsequently, subsamples were filtered on to 0.2 and 0.8 μm black polycarbonate filters for bacterial and protist quantification respectively (all Whatman, GE Healthcare Europe GmbH). All filters were stained for 2 min with a 0.01 mg/mL solution of 4′,6-diamidino-2-phenylindole (DAPI) and quantified under a BX-50 epifluorescence microscope at 600X or 1000X using filter sets U-FUW for DAPI and U-FBNA for chlorophyll autofluorescence and FLB fluorescence (all Olympus Co., Japan). A minimum of 200 cells or 150 randomized counting fields were quantified and examined for the presence of ingested FLB.

The number of ingested FLB per individual (FLB ind^−1^) was determined for the whole population and for the fraction of feeding phytoflagellates (determined for that day with LyTG as described above). To account for potential differences in inoculated FLB between replicates, FLB per individual was transformed to clearance per individual (nl indiv^−1^). Bacterivory was considered below detection if no significant differences were observed in the clearance per individual between t 0 and t 30 min (*t*-test, *P* > 0.05). Where bacterivory was detectable, the hourly clearance rate (nl ind^−1^ h^−1^) for the whole population and for feeding phytoflagellates was obtained by linear regression of the exponential uptake phase (Sherr et al., 1987). Clearance rates were transformed to ingestion (bacteria ind^−1^ h^−1^) and bacterivory rates (bacteria mL^−1^ h^−1^).

### Measurements of chlorophyll a concentration (chl a), primary production and cellular carbon content

To determine Chl *a* concentrations (pg Chl *a* mL^−1^), 10 mL were taken from each experimental flask and filtered on to GF/F filters (Whatman). These were submerged in 96% ethanol for up to 24 h and stored at −80°C until measurement on a fluorometer (Trilogy, Turner Designs CA, USA). Values were transformed to pg Chl *a* cell^−1^. Primary production was measured as previously described (Skovgaard et al., 2000). Briefly, two 2 mL aliquots from each experimental flask were transferred to 20-mL glass scintillation vials, and 20 μL of NaH^14^CO_3_ stock solution were added to each vial (specific activity = 100 μCi mL^−1^; Carbon-14 Centralen, Denmark). One vial of each pair was incubated for 3 h under the same conditions as the experimental flask, and the other vial was kept in complete darkness. After incubation, a 100 μL sub-sample was withdrawn from each vial and added to a new vial containing 200 μL phenylethylamine for measurements of specific activity. The remaining 1.9 mL were acidified with 10% glacial acetic acid in methanol, and evaporated overnight at 60°C to remove all inorganic carbon. The residue in the vial was re-dissolved in 2 mL Milli-Q water before adding 10 mL of scintillation cocktail (Insta-Gel Plus, Packard, USA). All vials were vigorously shaken and then analyzed using a liquid scintillation counter (Tri-Carb 2910 TR, Perkin-Elmer). Primary production (PP) (pg C cell^−1^ h^−1^) was calculated as follows:

$$\begin{array}{l} \text{PP}=\text{DPM}\times \text{DIC}{/}^{14}\text{C}\times \text{t}\times \text{N} \end{array}$$

where DPM is disintegrations min^−1^ mL^−1^ in the light value (corrected for dark), DIC is the concentration of dissolved inorganic carbon [pg C mL^−1^; determined simultaneously using a total organic carbon analyzer (TOC-L, Shimadzu, Japan)], ^14^C is the specific activity (disintegrations min^−1^ mL^−1^), t is the incubation time (in h), and N is the number of cells mL^−1^ at the time of sampling.

To determine cellular C content, unfiltered and GF/C (Whatman)-filtered samples from each experimental flask were measured on a total organic carbon analyzer (TOC-L, Shimadzu, Japan; all material was combusted at 450°C prior to use) and the pg C cell^−1^ was calculated as:

$$\begin{array}{l} \text{Cellular C content}=\left({\text{C}}_{\text{unfiltered}}-{\text{C}}_{\text{filtered}}\right)/\text{N} \end{array}$$

where C is total organic carbon measured (in pg mL^−1^) and N is the number of cells mL^−1^ at the time of sampling.

To estimate the balance between primary production and C required for division, the C produced during the doubling time on day 7 was divided by the cellular C content.

### Statistical tests

Significant differences between data sets were detected using two-tailed *t*-tests run on the SPSS statistics software (IBM Denmark ApS).

## Results

### pH, nutrient and bacterial concentrations

Tests conducted prior to the experiments indicated that the three algal strains grew well at the pH range observed during the main experiments (Supplementary Figure 1) and the follow up experiments (pH < 9; data not shown). N and P concentrations were low in the sea water used as the media base [6–10.2 μM N (from NO_3_ and NH_4_) and 0.55–1 μM P (from PO_4_)]. Nutrient carry over with the protist inoculum was not measured, but N and P concentrations measured in NMa on day 3 and 7 indicate that both were rapidly depleted (Range for all three experiments—Day 3: 2.7–16.4 μM N and 0.1–0.4 μM P; day 7: 1.1–1.7 μM N and 0.2–0.3 μM P). Concentrations for all other treatments remained high (range on day 7: 230–550 μM N and 12.2–26.2 μM P). Bacterial abundance at the start of the experiments was 1.82 ± 0.49 × 10^5^ cell mL^−1^ for *N. rotunda* Exp. 2, 4.04 ± 1.1 × 10^6^ cell mL^−1^ for *N. rotunda* Exp. 3, 2.05 ± 0.83 × 10^5^ cell mL^−1^ for *N. pyriformis* and 1.76 ± 0.57 × 10^5^ cell mL^−1^ for *I. galbana* (average and standard deviation for all treatments). Bacterial concentration increased throughout the experiments for all treatments reaching maximum values of 8.2 × 10^6^ cell mL^−1^ for *N. rotunda*, 4.2 × 10^6^ cell mL^−1^ for *N. pyriformis* and 9.9 × 10^6^ cell mL^−1^ for *I. galbana* (Supplementary Figure 5).

### Phytoflagellate abundance, growth, primary production and cellular C content

**Figure 1:**
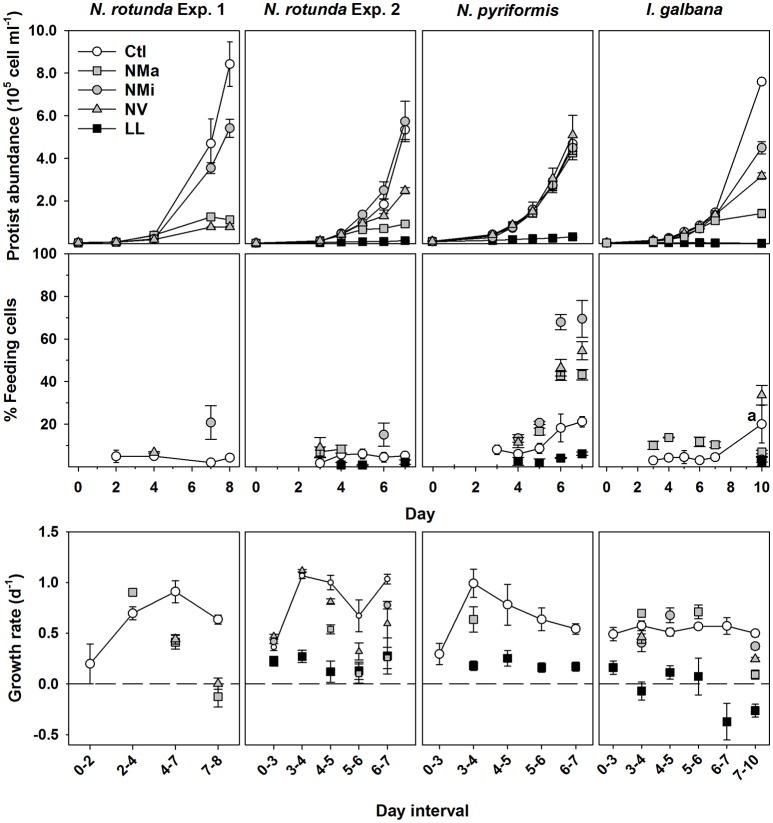
Changes in phytoflagellate abundance, percentage of feeding cells and growth rate for *N. rotunda* Exp. 1 and 2; and the *N. pyriformis* and *I. galbana* experiments. In the plots for percentage of feeding cells and growth rate all time points for Ctl are plotted, while for the treatments only the time points with a significant difference to Ctl are plotted (*t*-test; *P* < 0.05). a, at this time point only duplicate values were available for the control and statistics were not possible. Treatments – control (Ctl), and media without the addition of macronutrients (Nma), micronutrients (NMi) or vitamins (NV).

Cross-comparisons between phytoflagellate flow-cytometric and microscopic enumeration indicated both methods were overall highly comparable, though *N. rotunda* cell counts tended to be slightly higher when quantified via microscopy at high cell concentrations (Supplementary Figure 3). All three species grew well in Ctl, and exhibited low (*Nephroselmis* spp.) or no growth (*I. galbana*) in LL (Figure 1). Growth in the other treatments varied strongly between species (Figure 1). Phytoflagellate abundance in *N. rotunda* Exp. 3 was comparable to results from Exp. 1 and 2. The initial algal concentration in Exp. 3 did not differ significantly between treatments (*t*-test, *P* > 0.05; 3.3 ± 0.1 × 10^3^ cell mL^−1^ for all three treatments), while on day 6, both NMa and NMi showed significantly lower algal abundance than the control (*t*-test, *P* < 0.05; 0.03 ± 0.0, 1.2 ± 0.2 and 2.2 ± 0.5 × 10^6^ cell mL^−1^ respectively).

Significant reductions in cellular primary production rates, and Chl *a* concentrations with respect to Ctl were observed for all species under LL, and for *N. rotunda* under NMa and NMi (Table 3; *t*-test, *P* < 0.05). In NV neither *N. rotunda* cellular primary production nor Chl *a* concentration were significantly lower than in Ctl, but the ratio between the two was significantly lower (*t*-test, *P* < 0.05). For all other species and treatments no significant differences were observed to the Ctl (*t*-test, *P* > 0.05). Likewise, cellular C content was more variable for N. rotunda than for the other two species (Table 3). Finally, estimates of the balance between primary production and C requirements for growth indicate that for all 3 species and treatments (excepting LL), considerably more carbon was produced than was required to double cellular biomass.

**Table 3:** Cellular primary production (PP), cellular Chl a concentration, PP/Chl *a* and cellular C content for the 3 algal strains.

	**Chl a (pg cell**^**−1**^**)**		**PP (pg C cell**^**−1**^ **h**^**−1**^**)**		**PP/Chl a**		**Carbon content (pg cell**^**−1**^**)**		**C produced/C required for division**
	**av**	***sd***	**av**	***sd***	**av**	***sd***	**av**	***sd***	
***N. rotunda*** **Exp. 2**									
Ctl	0.31	0.03	1.12	0.19	3.58	0.55	4.7	1.0	3.9
NMa	0.21^*^	0.03	0.28^*^	0.02	1.36^*^	0.26	2.2^a^	0.4	8.4
NMi	0.23^*^	0.04	0.71^*^	0.13	3.00	0.19	2.5^a^	0.1	5.5
NV	0.42	0.07	0.78	0.14	1.87^*^	0.14	7.4^*^	0.1	3.0
LL	0.60^*^	0.07	0.05^*^	0.02	0.08^*^	0.04	–	–	–
***N. pyriformis***									
Ctl	0.17	0.01	0.97	0.08	5.65	0.16	4.2	0.9	7.0
NMa	0.23^*^	0.01	1.47	0.39	6.41	1.92	4.7	0.6	12.0
NMi	0.20	0.04	0.85	0.23	4.50	2.10	3.27	0.5	8.8
NV	0.15	0.01	1.03	0.45	6.67	2.66	3.8^a^	0.8	7.2
LL	0.60^*^	0.08	0.11^*^	0.08	0.17^*^	0.11	–	–	–
***I. galbana***									
Ctl	0.24	0.01	1.05	0.09	4.28	0.36	6.2^a^	0.6	4.9
NMa	0.27	0.04	1.67	0.44	6.34	2.03	7.9	2.1	9.3
NMi	0.26	0.02	0.98	0.26	3.71	0.78	5.7	0.9	4.9
NV	0.29	0.03	1.15	0.50	3.99	1.71	4.3	1.1	9.5
LL	0.53^*^	0.10	0.11^*^	0.09	0.20^*^	0.11	–	–	–

* Significant differences to Ctl.

*– Could not be determined accurately due to very low algal abundance*.

a *Statistics could not be carried out since only 2 replicates were available*.

### Phytoflagellate feeding

Cells containing food vacuoles, and therefore presumed to be feeding, were found in all treatments, though percentages tended to be <10% for LL and most Ctl time-points (Figure 1). *N. rotunda* feeding cells generally remained below 7% of total abundance, irrespective of treatment, though a brief significant increase was observed for NMi on days 7 and 6 for Exp. 1 and 2 respectively (Figure 1; values for Exp. 3 on day 6—Ctl: 2.5 ± 0.7%; NMa: 1.1 ± 0.3%; NMi: 2.0 ± 0.5%). *N. pyriformis* in all treatments and *I. galbana* in NMa and NV responded to nutrient limitation by significantly increasing percentages of feeding cells with respect to Ctl, reaching maximum values of 79 and 37% respectively (*t*-test, *P* < 0.05).

**Figure 2:**
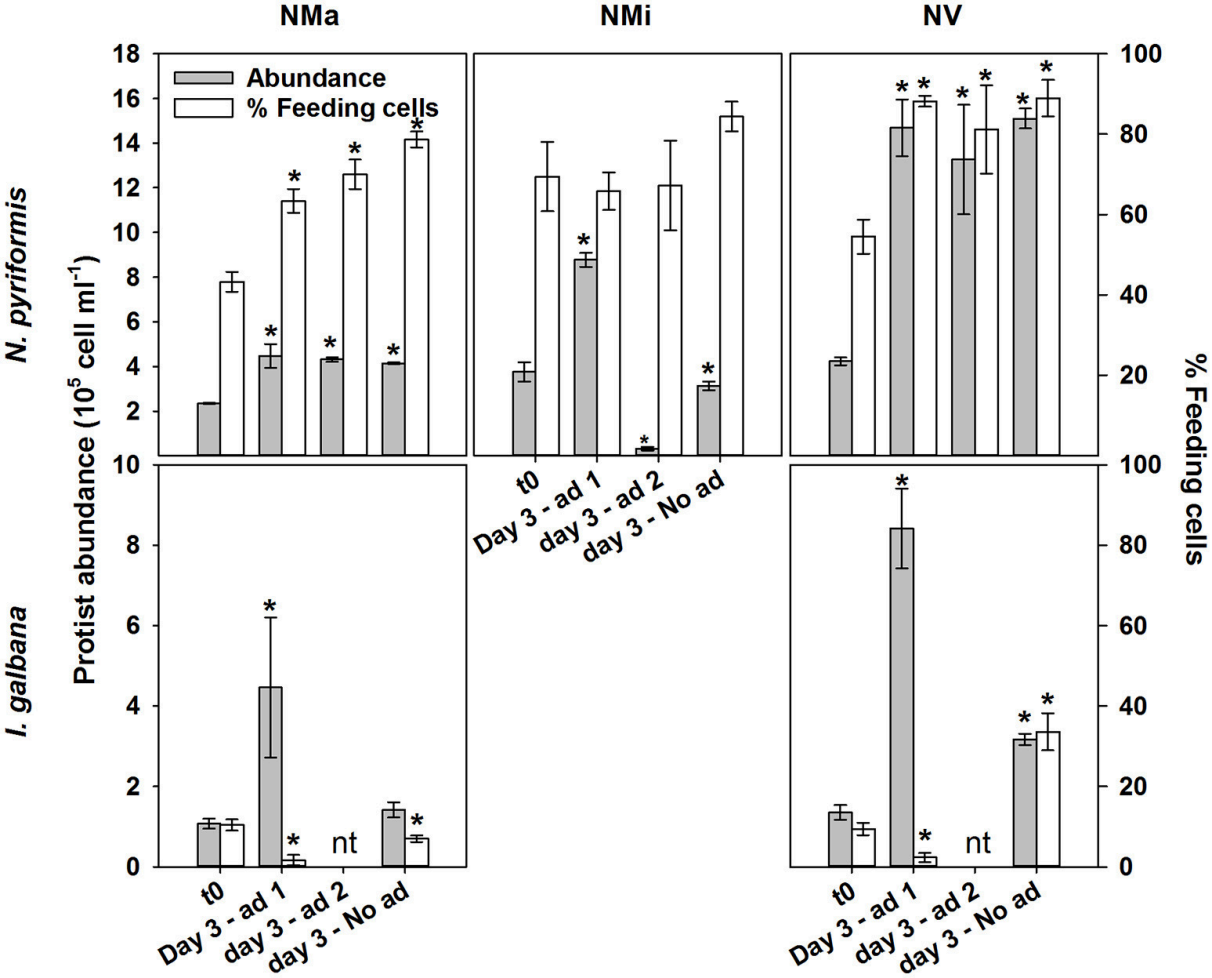
Follow up experiments ascertaining the regulatory role of the “limiting” substrate on algae growth and feeding. These were conducted with the *N. pyriformis* and *I. galbana* treatments NMa, NMi and NV. Ad 1, addition 1 - addition of the “limiting” solution (e.g., vitmains to the NV treatment); Ad 2, addition 2 - addition of the other solutions (e.g., macro- and micronutrients to the NV treatment); No ad, unamended treatment; nt, not tested. ^*^Indicates significant differences to t0 (*t*-test; *P* < 0.05).

**Figure 3:**
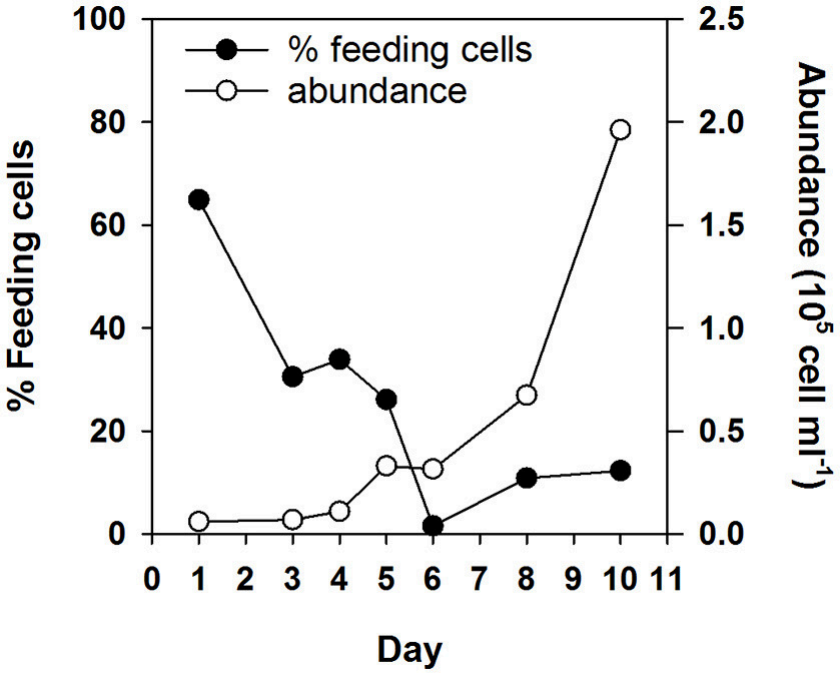
Change in phytoflagellate abundance and percentage of feeding cells over time for a starved *N. pyriformis* culture after being re-inoculated into nutrient replete media.

Follow up experiments confirmed limitation by the “limiting” substrate in *N. pyriformis* NMi and *I. galbana* NMa and NV (Figure 2; significant increase in phytoflagellate abundance in Addition 1, but not in Unamended and Addition 2 treatments). However, co-limitation was likely occurring by that point in *N. pyriformis* NMa and NV (no significant differences in the increase of phytoflagellate abundance between treatments). Addition of the “limiting” substrate led to a significant decrease in the percentage of feeding *I. galbana* cells (*t*-test, *P* < 0.05), but the percentage of feeding *N. pyriformis* cells remained relatively constant (Figure 2). In a subsequent test with *N. pyriformis*, after transfer of a starved culture to nutrient-replete media, 6 days were required for percentages of feeding cells to decrease <10% (Figure 3).

**Figure 4:**
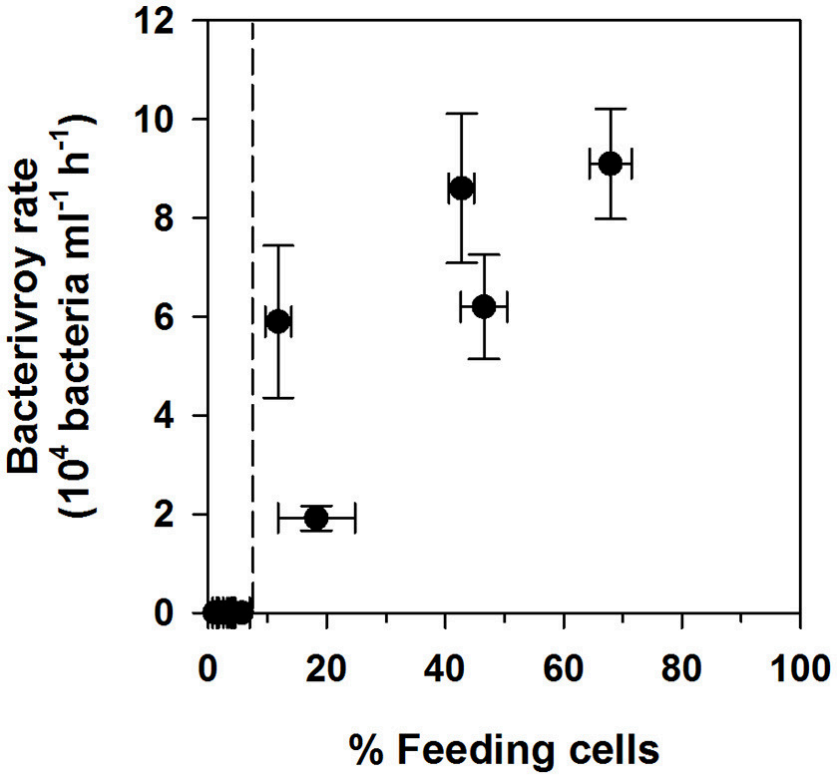
Comparison of measured bacterivory rates and percentages of feeding cells. Data has been pooled for all three algal strains. The dashed line indicates the observed threshold for bacterivory rate detection.

Bacterivory rates were only detectable when the percentage of feeding cells exceeded 6–10% (Figure 4 and Table 4). Where bacterivory was detectable, ingestion and clearance rates calculated for the whole community or only for feeding cells showed an up to 11-fold significant difference. *N. pyriformis* was the only algal strain with detectable bacterivory in more than one treatment. This revealed that the comparison between Ctl and the treatments was also impacted by how ingestion rates were calculated. Bacterivory rates and clearance and ingestion rates calculated for the whole community were all significantly higher in the treatments than in Ctl. However, when calculated solely for feeding cells, ingestion rates and all clearance rates except that for NMi, did not differ from Ctl.

**Table 4:** Bacterivory rates and ingestion and clearance rates determined for all phytoplankton cells and exclusively for feeding cells.

	**% Feeding cells**		**Ingestion rate (bacteria ind**^**−1**^ **h**^**−1**^**)**				**Clearance rate (nl ind**^**−1**^ **h**^**−1**^**)**				**Bacterivory rate (10**^**4**^ **b mL**^**−1**^ **h**^**−1**^**)**	
			**Total cells**		**Feeding cells**		**Total cells**		**Feeding cells**		
	**av**	***sd***	**av**	***sd***	**av**	***sd***	**av**	***sd***	**Av**	***sd***	**av**	***sd***
***N. rotunda*** **Exp. 3**												
Ctl	2.5	0.7	b.d.									
NMa	1.1	0.3	b.d.									
NMi	2.0	0.5	b.d.									
***N. pyriformis***												
Ctl	18.2	6.5	0.2 ^∧^	0.0	1.6 ^∧^	0.4	0.1 ^∧^	0.0	0.8 ^∧^	0.2	1.9	0.3
NMa	42.7	2.1	0.9 ^*^^∧^	0.3	1.9 ^∧^	0.7	0.4 ^*^^∧^	0.1	0.9 ^∧^	0.2	8.6^*^	1.5
NMi	67.9	3.6	0.8^*^^∧^	0.1	1.2 ^∧^	0.1	0.3^*^^∧^	0.0	0.5 ^*^^∧^	0.0	9.1^*^	1.1
NV	46.5	3.9	0.6 ^*^^∧^	0.1	1.2 ^∧^	0.1	0.2 ^*^^∧^	0.0	0.5 ^∧^	0.1	6.2^*^	1.1
LL	4.1	0.3	b.d.									
***I. galbana***												
Ctl	3.1	1.4	b.d.									
NMa	11.8	2.2	1.0 ^∧^	0.1	11.5 ^∧^	1.5	0.7 ^∧^	0.1	5.7 ^∧^	1.6	5.9	1.5
NMi	5.6	1.3	b.d.									
NV	3.8	0.3	b.d.									
LL	3.8	0.3	b.d.									

* Significant differences with control (t-test P < 0.05).

∧ *Significant differences between calculations based on total abundance and feeding cells (t-test P < 0.05)*.

*b.d. below detection*.

## Discussion

### Methodological considerations—fraction of feeding cells and bacterivory rates

In the present study a detection threshold for bacterivory rates in cultured small phytoflagellates could be determined for the first time, at around 6–10% of feeding cells (Figure 4 and Table 4). Whether this threshold also applies to other species could be an important consideration, especially for those, such as the green algae *Cymbomonas* sp., that appear to feed infrequently (Maruyama and Kim, 2013; Burns et al., 2015). Levels of feeding cells <10% have been detected previously for broad phytoplankton size classes in the field (Anderson et al., 2017) and could explain the absence of detectable bacterivory rates for certain phytoflagellate groups in culture (e.g., see Table 1) and in the field (Moorthi et al., 2009; Unrein et al., 2013).

Overall, under no circumstances were 100% of cells feeding in the present study, and fractions of feeding cells were always below 40% for both *I. galbana* and *N. rotunda* (Figures 1, 2). Similar observations have been made for the bacterivorous chrysophyte *Ochromonas danica* (Aaronson, 1974) and the haptophyte *Prymnesium parvum* when fed with a cryptophyte (Carvalho and Granéli, 2006). A large fraction of non-feeding phytoflagellate cells could thus be a common phenomenon, perhaps due to a fraction of cells in division and micro-scale nutrient patchiness, leading cells to experience different levels of limitation. In the field, this phenomenon has been pinpointed as a significant potential source of bias in the determination of phytoplankton community ingestion rates, due to the inherent assumption in routinely used calculations that all cells are feeding (Weisse et al., 2016; Anderson et al., 2017). In the present study ingestion rates calculated solely for feeding cells were always significantly higher than those calculated for the whole population, differing by a factor of 1.5–11 (Table 4). In addition, for *N. pyriformis*, restricting calculations from all cells to feeding cells altered the perceived mixotrophic response, shifting from increased ingestion rates under nutrient-limited conditions, to constant rates, with changes in total bacterivory being rather due to changes in the number of feeding cells. It should be noted that the bacterivory rates determined here need to be considered as estimates, due to the well-known potential issue of protists selectively rejecting surrogate prey particles, such as FLB (e.g., Landry et al., 1991). However, these results overall confirm that assuming all cells are feeding can also critically bias both calculated ingestion rates and the perceived mixotrophic response for cultured phytoflagellates. The application of acidotropic probes in concert to “classic” techniques to determine bacterivory is a simple and rapid technique to correct this problem (Anderson et al., 2017).

Finally, it should be noted that the prey base consisted of the bacterial community naturally co-occurring with each SP strain. The premise for this choice, rather than a standardized prey for all experiments, was based on the fact that each SP strain should be acclimated to the co-occurring bacterial community and adapted to utilize it when needed. As we did not analyse bacterial community composition, we cannot discard that differences in the edibility of the bacterial prey base could have influenced the results obtained for the three SP strains. However, strong differences in ingestion rates were also observed when offered FLB (which were the same for all SP species) and the differing patterns observed between the three species in algae growth and percentage of feeding cells are more drastic than would be anticipated based on food-quality alone. Thus, obtained results are in all likelihood primarily due to physiological/metabolic differences between the three SP strains (discussed in detail below).

### Mixotrophic feeding and growth of the three algal strains

In the present study, the mixotrophic response of the chlorophyte genus *Nephroselmis* and the haptophyte genus *Isochrysis* were assessed for the first time. All three tested species were capable of phagocytosis (Figure 1). However, none fed to any relevant degree under low light conditions (percentages of feeding cells were always <7% and generally <3); indicating that the three species are likely obligate phototrophs. This pattern has been observed previously, e.g., in the chrysophyte *Dinobryon* sp. (Caron et al., 1993; Liu et al., 2016) and a considerable number of algivorous red-tide dinoflagellates (Hansen, 2011); and has been linked to the obligate use of photosynthesis to obtain carbon, with prey primarily serving as a source of nutrients (Caron et al., 1993). In the present study, measured primary production in all treatments except LL was considerably in excess of the carbon required for division (Table 3), confirming that all three algal strains likely employed prey primarily as a source of nutrients. However, when subjected to nutrient limitation, the three species showed very different levels of feeding and mixotrophic growth.

*N. pyriformis* could fully compensate macronutrient, micronutrient and vitamin limitation by feeding, achieving equal growth rates to the control (Figures 1, 2; it should be noted that in NMa and NV the algae was co-limited at least toward the end of the experiment). To our knowledge, this is the first measurement of chlorophyte mixotrophic growth under nutrient limited conditions, and is in stark contrast to the classical view of green-algae as primarily non-feeders. In a comparable study, the polar chlorophytes *Pyramimonas tychotreta* and *Micromonas* sp. showed higher ingestion rates under low nutrient conditions (growth was not measured) (McKie-Krisberg and Sanders, 2014; McKie-Krisberg et al., 2015). Thus, bacterivory to counter nutrient limitation could be a wide-spread, though not universal, strategy among green-algae.

*N. rotunda*, in contrast to *N. pyriformis*, could not use bacterivory to counter nutrient limitation despite clear indications it was able to form food vacuoles. Percentages of feeding cells remained overall very low, with only a transient increase to around 20% in NMi; and growth decreased (NMi) or ceased (NMa and NV) as the phytoflagellate became limited for the different nutrients tested (Figure 1). Whether *N. rotunda* is missing key genes involved in the “activation” of feeding and/or the digestion and assimilation of prey is a very interesting field for future studies. Further, the strong intra-genus contrast in mixotrophic growth potential observed here for *Nephroselmis* spp. has previously only been seen within the chrysophyte *Ochromonas* spp., which can range from obligate phototrophs to primarily heterotrophic species (Keller et al., 1994; Liu et al., 2016; Terrado et al., 2017); and, to a lesser degree, among the red-tide dinoflagellate *Karlodinium* spp. (Berge et al., 2008; Calbet et al., 2011). Further studies should assess whether such differences are common or isolated events, and this variability should be considered when predicting trophic status of a novel strain or OTU based solely on genetic affinity.

The haptophyte *I. galbana* fed when subjected to macronutrient and vitamin limitation (Figures 1, 2). However, in contrast to *N. pyriformis*, the mixotrophic response resembled a survival mechanism rather than a growth strategy, with bacterivory only transiently compensating for nutrient limitation (NMa) or sustaining significantly lower growth rates than in in the control (NV). Micronutrient limitation did not appear to trigger feeding, but the decrease in growth with respect to the control was small, indicating limitation may just have started. Feeding which does not translate into significant growth has also been observed for strains of the haptophyte *Prymnesium parvum* and the chlorophyte *Cymbomonas* sp. when light limited, with prey postulated as a temporary means for cell maintenance (Brutemark and Granéli, 2011; Maruyama and Kim, 2013; Liu et al., 2016). Thus, the use of mixotrophy for short-term survival could be a common strategy, with important implications for phytoflagellate biomass production and, thus, the impact of mixotrophic feeding on the transfer of carbon and energy through marine food webs. As an example, under macronutrient limitation in the present study, an equivalent prey consumption by *N. pyriformis*, which used mixotrophy for growth, and *I. galbana*, which appeared to use mixotrophy for survival, (Table 4) resulted in significantly different outcomes in terms of phytoflagellate growth.

As an additional interesting aspect, *N. pyriformis* and *I. galbana* not only showed distinct differences in mixotrophic growth potential, but also in the speed at which they could respond to changes in ambient nutrient concentrations. Macronutrient- and vitamin-starved *I. galbana* cells had almost entirely stopped feeding three days after limiting conditions ceased (Figure 2; <3% of feeding cells), while a lag of almost a week was observed for starved *N. pyriformis* (Figures 2, 3). It should be noted that due to the long-time scales used in this study, the “feeding signal” observed will almost certainly be due to the continued formation of new food vacuoles and not the detection of “old” food vacuoles that are still being digested (González et al., 1990; Boenigk et al., 2001; Anderson et al., 2017). In a similar study under P-limiting conditions, the haptophyte *Prymnesium parvum* (formerly *P. pateliferum*) decreased its P-limitation-linked hemolytic activity within 24 h of PO_4_ or bacterial prey addition (Legrand et al., 2001). Further studies are needed to determine whether these differences in trophic flexibility are phylogenetic, the result of metabolic and energetic trade-offs at an individual species level (Raven, 1997), and/or the consequence of adaptation to environments with differing nutrient dynamics.

The variations in the mixotrophic response of *I. galbana* and *N. rotunda* when limited by different substrates may reflect a differential capacity for assimilating distinct substrates from prey. Feeding patterns in other phytoflagellate strains have at least been partly explained by auxotrophy for specific complex macromolecules (Kimura and Ishida, 1985; Sanders and Porter, 1988); the loss of certain genes, such as nitrate/nitrite reductase in *Ochromonas* (Liu et al., 2016); or preferential assimilation of specific substrates from different prey (Liu et al., 2015). An additional interesting aspect is the effect of nutritional history on the mixotrophic response of algal strains. In contrast to the present study, (Anderson et al., 2017) found no feeding in the exact same *Nephroselmis* strains tested here under nutrient and light replete conditions (Table 1). The main difference between the two studies was the nutritional history of the pre-culture, which was serially starved in the present study and maintained in nutrient replete media in Anderson et al. (2017). A very similar pattern was observed with the haptophyte *Imantonia* sp. (Table 1), indicating that past starvation could potentially “prime” a population to respond rapidly to a new nutrient limitation by maintaining low levels of feeding cells.

Bacterivory rates obtained in this study were relatively high but in the range detected for other chlorophyte and haptophyte groups (Table 4; Legrand et al., 2001; Unrein et al., 2013; McKie-Krisberg and Sanders, 2014; McKie-Krisberg et al., 2015). This was not reflected in the bacterial abundance (Supplementary Figure 5), which remained relatively constant between treatments. However, as bacterial production and composition were not measured, it cannot be discarded that selective predation, potential differences in the quantity and quality of organic matter released by the algae in different trophic modes, and limitation also among the bacteria for the target substrates (e.g., micronutrients in the NMi treatment) had a differential impact on bacterial growth (Posch et al., 1999; Hale et al., 2017; Seymour et al., 2017). Overall, it should be noted that these experiments were conducted with very low bacterial concentrations (never exceeding 10^7^ cell mL^−1^), with the aim of observing SP feeding and mixotrophic growth at bacterial cell-counts that would realistically be found in nutrient limited marine systems (e.g., Gasol et al., 2002). It thus cannot be excluded that *N. rotunda* and *I. galbana* would show a different mixotrophic response at higher, but less environmentally relevant, bacterial concentrations. However, it should be highlighted that bacterivory significantly contributed to *N. pyriformis* growth even at bacterial concentrations below 5 × 10^6^ cell mL^−1^ (Supplementary Figure 5). Thus, even under low bacterial standing stocks, equivalent to those found in oligotrophic open oceans, mixotrophic bacterivory can significantly contribute to the nutritional needs of marine phytoflagellates.

Overall, the present study revealed strong differences in the feeding, mixotrophic growth potential and trophic flexibility of the tested species. These results point to the possibility that, beyond previous classification systems for mixotrophs based on substrate uptake from prey (Stoecker, 1998), “survival” and “growth” mixotrophy strategies could exist. The “survival strategy,” exemplified here by nutrient-limited *I. galbana*, would be characterized by low levels of feeding coupled to the ability to rapidly revert to being a strict phototroph. This mixotrophy strategy could be of advantage in environments where nutrient limitation is transient, and could serve as a way to minimize the costs thought to be associated with maintaining both photosynthetic and feeding machineries (Raven, 1997). Similar observations have been made previously for the haptophyte *Prymnesium parvum* and the chlorophyte *Cymbomonas* sp. with feeding postulated as a means to withstand low light conditions (Brutemark and Granéli, 2011; Maruyama and Kim, 2013; Liu et al., 2016). Conversely, the “growth strategy,” exemplified here by *N. pyrifomis*, would be characterized by high mixotrophic growth efficiency with a potential trade-off in a lower trophic flexibility (it took approximately a week for the *N. pyriformis* population to revert to being primarily phototrophic). This mixotrophy “growth strategy” seems suited to environments where SP will be subjected to prolonged periods of nutrient limitation, favoring a higher investment in the feeding machinery. Further studies are needed on a broad range of phylogenetically and geographically diverse SP to be able to confirm these speculations. However, it is important to note that the survival vs. growth mixotrophy strategies will lead to strong differences in prey-to-algal biomass conversion efficiency and thereby the role mixotrophy plays in linking bacteria to higher levels in the food chain. Thus, the existence of these two strategies could imply that mixotrophy plays different roles in contrasting environments, with important implications for the global role of mixotrophy in the transfer of energy and matter through marine ecosystems (Mitra et al., 2013).

## Author contributions

RA, SC, and PH designed the experiments. RA conducted the experiments, analyzed the data and wrote the manuscript. SC and PH contributed to manuscript preparation.

### Conflict of interest statement

The authors declare that the research was conducted in the absence of any commercial or financial relationships that could be construed as a potential conflict of interest.
