# Supplementary figures and images for: Mixotrophy in Chlorophytes and Haptophytes—Effect of Irradiance, Macronutrient, Micronutrient and Vitamin Limitation
Source: Front Microbiol. 2018 Jul 31;9:1704. doi: 10.3389/fmicb.2018.01704 (PMC6080504; doi:10.3389/fmicb.2018.01704)

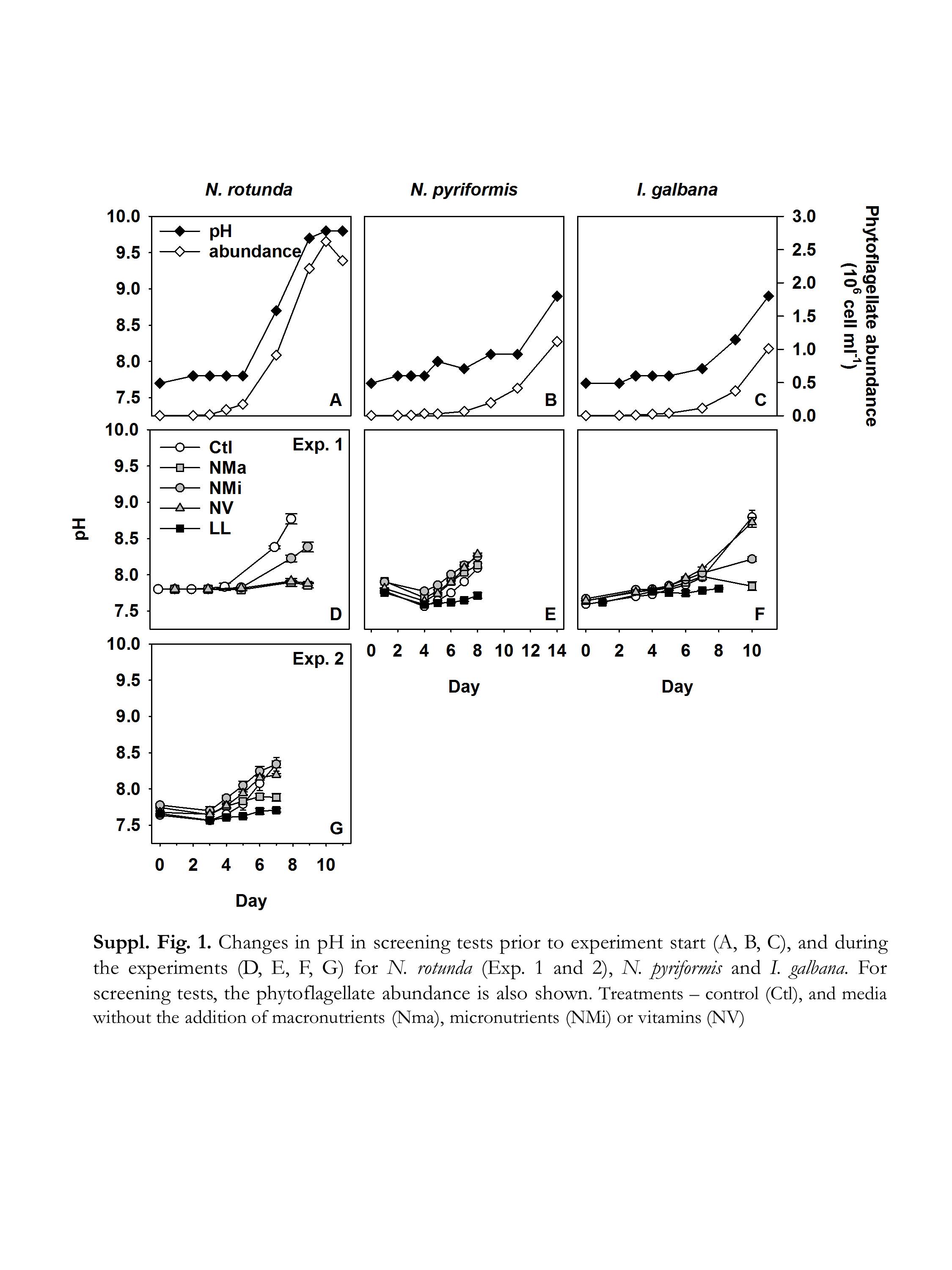

Supplement: Supplementary file 1 [file Image_1.JPEG]

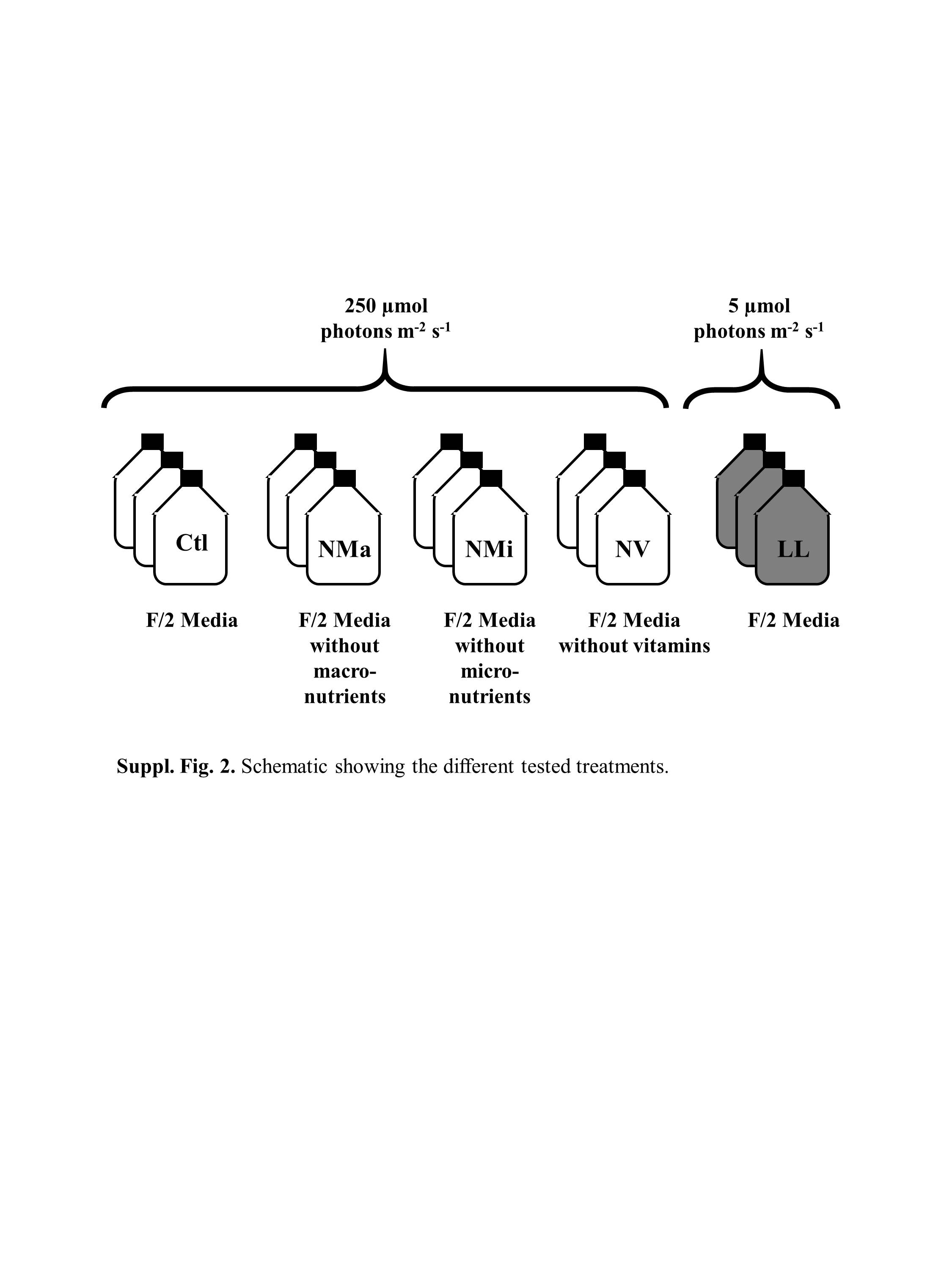

Supplement: Supplementary file 2 [file Image_2.JPEG]

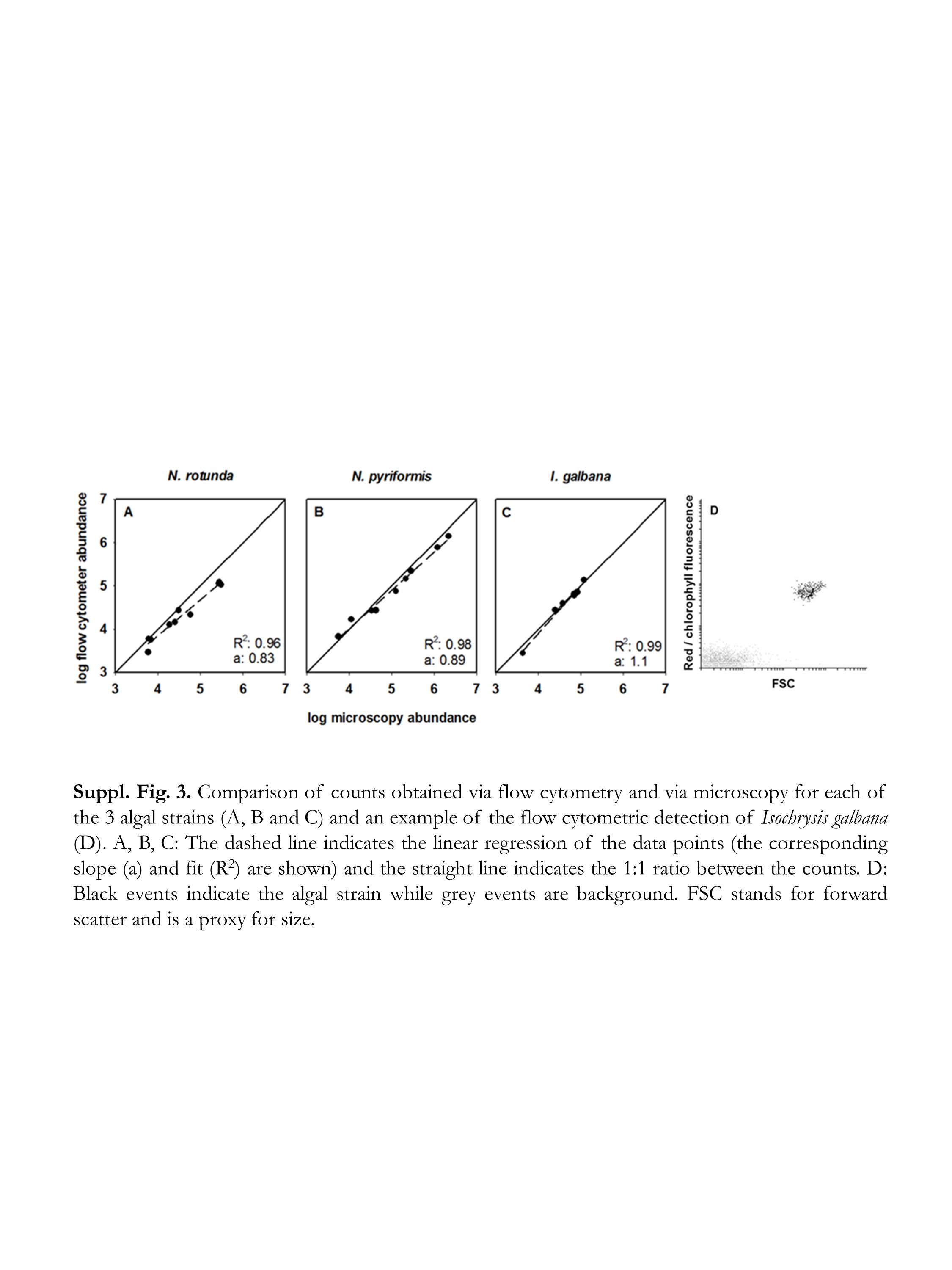

Supplement: Supplementary file 3 [file Image_3.JPEG]

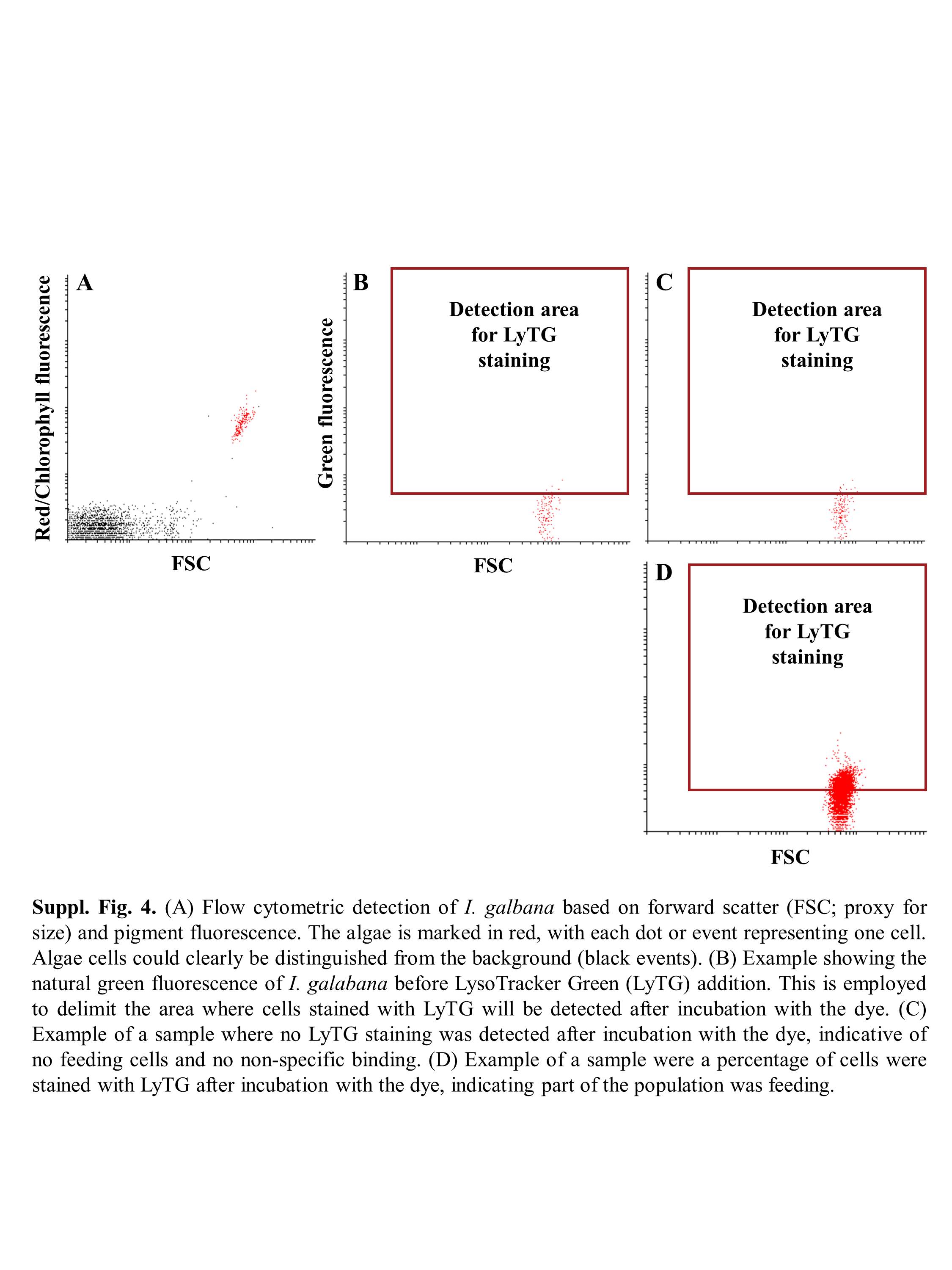

Supplement: Supplementary file 4 [file Image_4.JPEG]

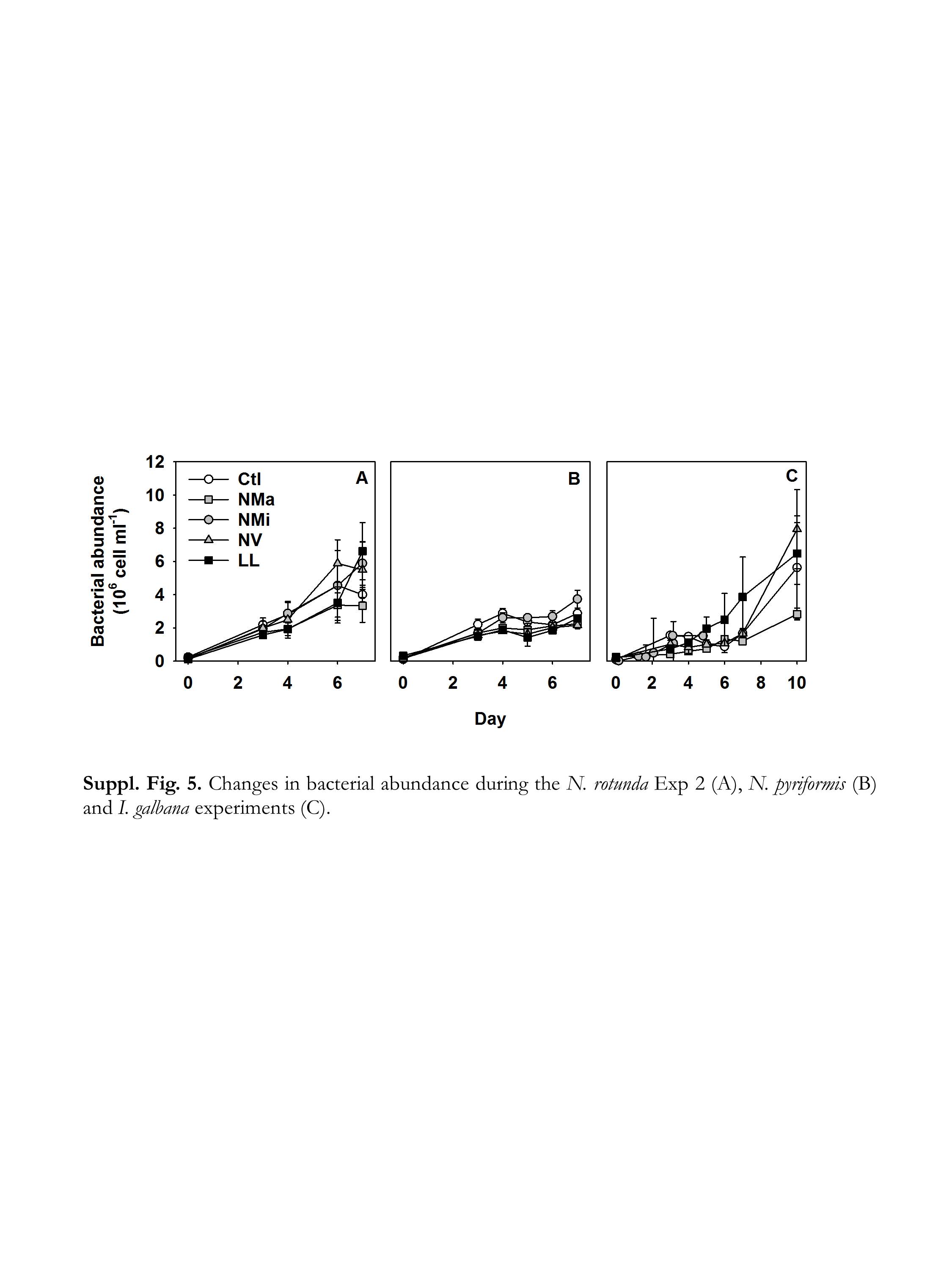

Supplement: Supplementary file 5 [file Image_5.JPEG]
